# TLR agonists induce sustained IgG to hemagglutinin stem and modulate T cells following newborn vaccination

**DOI:** 10.1038/s41541-022-00523-8

**Published:** 2022-08-29

**Authors:** Elene A. Clemens, Beth C. Holbrook, Brendan McNeilly, Masaru Kanekiyo, Barney S. Graham, Martha A. Alexander-Miller

**Affiliations:** 1 Department of Microbiology and Immunology, Wake Forest University School of Medicine, Winston-Salem, USA; 2 Vaccine Research Center, NIAID, NIH, Bethesda, USA

**Keywords:** Adjuvants, Inactivated vaccines

## Abstract

The newborn immune system is characterized by diminished immune responses that leave infants vulnerable to virus-mediated disease and make vaccination more challenging. Optimal vaccination strategies for influenza A virus (IAV) in newborns should result in robust levels of protective antibodies, including those with broad reactivity to combat the variability in IAV strains across seasons. The stem region of the hemagglutinin (HA) molecule is a target of such antibodies. Using a nonhuman primate model, we investigate the capacity of newborns to generate and maintain antibodies to the conserved stem region following vaccination. We find adjuvanting an inactivated vaccine with the TLR7/8 agonist R848 is effective in promoting sustained HA stem-specific IgG. Unexpectedly, HA stem-specific antibodies were generated with a distinct kinetic pattern compared to the overall response. Administration of R848 was associated with increased influenza-specific T follicular helper cells as well as Tregs with a less suppressive phenotype, suggesting adjuvant impacts multiple cell types that have the potential to contribute to the HA-stem response.

## Introduction

Attempts to develop a vaccine conferring multi-season protection from influenza A virus (IAV) have long been hindered by viral antigenic shift and drift that allows the virus to escape from immune recognition^1–3^. To overcome this hurdle, there has been a recent focus on the elicitation of antibodies (Ab) targeting conserved viral structures. The most extensively studied of these is the stem region of the hemagglutinin (HA) surface protein, which is responsible for viral attachment and fusion^4–8^. While the HA stem is highly conserved across strains, it is not highly immunogenic, resulting in preferential generation of Ab responses to variable epitopes on the HA head^9–11^. Although this phenomenon of Ab immunodominance to IAV epitopes has been reported, the mechanisms driving it are poorly understood^12–14^.

Newborns are a population that is particularly vulnerable to severe disease following IAV infection^15,16^. In general, the newborn immune system favors tolerance over strong inflammatory and antiviral responses. While this is important to limit responses to environmental antigens and allow colonization by healthy microbiota^17,18^, it can lead to inadequate clearance of pathogens. Susceptibility to severe disease is further exacerbated by the lack of an effective IAV vaccine for infants under 6 month of age. The limited immune response to vaccination in young infants manifests as reduced production and maintenance of high-titer, high-affinity antibodies following antigen exposure^19^.

The generation of a high-quality antibody response relies on the effective coordination and interaction of a multitude of immunologic factors. While newborn B cells have been demonstrated to have intrinsic defects in activation, signaling, and maturation^20–26^, they are also impaired by diminished T cell help, immunosuppression, and structural changes in the lymphoid microenvironment that inhibit differentiation and survival^27–29^. In particular, newborns have impaired formation of germinal centers (GC) required for maturation and differentiation of memory B cells (MBC) and long-lived plasma cells (LLPC)^30–32^. In addition to structural constraints from stromal cells, alterations in the generation of T follicular helper cells (Tfh) and dendritic cell (DC) function have been identified as major barriers to effective GC formation and function^32–34^. Difficulties in mounting robust antibody responses are further exacerbated by increases in the number and activity of immunosuppressive Tregs during early life^35,36^.

The complex interaction of the many factors that regulate an antibody response makes this a highly dynamic process, especially since the acute effector and lasting MBC/LLPC responses are regulated by different processes. Similarly, the immunodominance hierarchy of Ab specificities to distinct epitopes evolves over time, suggesting that the selection criteria for dominant epitope specificities may shift and change over the course of the GC reaction^37^. At present, the mechanisms dictating antibody immunodominance are not well understood. One appealing model is that it immunodominance is, at least in part, the product of clonal competition within the GC and alleviating this competitive pressure can facilitate the expansion of subdominant clones, e.g., those specific for the HA-stem region. Indeed, recent studies have demonstrated that Ab responses to subdominant epitopes may be improved by increasing the accessibility of resources to clones that may be less competitive (e.g., increasing antigen dose or T cell help) as well as removing negative selection pressures (e.g., by reducing Treg suppression or targeted elimination of dominant clones)^38–42^. Unsurprisingly, many of the factors implicated in establishing an immunodominance hierarchy are involved in the normal maturation and differentiation of the antibody response^43,44^. This suggests overcoming subdominance of the HA stem and improving maintenance of the desired Ab response may be tightly entwined. It is not clear how the early life alterations in the function and development of immune cells involved in these processes may affect the dynamic immunodominance hierarchy.

Although a variety of strategies have been employed in attempts to elicit robust, persistent responses to conserved subdominant epitopes, the use of immune adjuvants is particularly appealing considering their ability to act upon a broad range of immune targets. Several reports in adult models have demonstrated that adjuvants can improve the quality and quantity of cross-protective antibody elicited by vaccination^31,45–50^. Using a nonhuman primate (NHP) model, we have previously shown that inclusion of the TLR agonist (TLRa) adjuvants flagellin and R848, either singly or in combination with a killed IAV vaccine, can improve the titer and consequently increased persistence of total IAV-specific Ab^51–54^. Further, we have demonstrated that newborn NHP are capable of producing a robust Ab response to the HA stem following infection with IAV^55^. We have also observed a beneficial effect of R848 on the early production of stem-specific antibody^56^. Here, we extend our studies into the elicitation of HA stem-specific antibodies in newborns by assessing the ability of flagellin and R848 to serve as adjuvants that can impact the kinetics and maintenance of a stem-specific Ab response as well as modulate immune populations that may regulate these responses. The NHP model used in these studies represent an extremely important translational model due to their immunologic, developmental, and physiologic similarities with humans^57^.

## Results

### TLRa adjuvants elicit a stem response upon vaccine boost

The generation of HA stem-specific antibody as a result of vaccination is challenging, given the subdominant nature of this response in the context of the HA molecule^10^. We have previously shown that inactivated influenza A/Puerto Rico/8/1934 virus (IPR8) admixed with flagellin (IPR8 + flg), directly conjugated to R848 (IPR8-R848), or the combination of both adjuvants (IPR8-R848 + flg) all increase IAV-specific IgG titers in newborn nursery-reared African green monkeys (AGM) as compared to non-adjuvanted IPR8^51–54^. Here, IPR8 with an inactive flagellin mutant (m229), which has a biologically inactive hypervariable region^58^, served as a non-adjuvanted vaccine. We have also demonstrated that prime and boost of nursery-reared AGM newborns with IPR8-R848 can improve both total and neutralizing titers of stem-specific antibody following live viral challenge at early points after vaccination^56^.

**Fig. 1:**
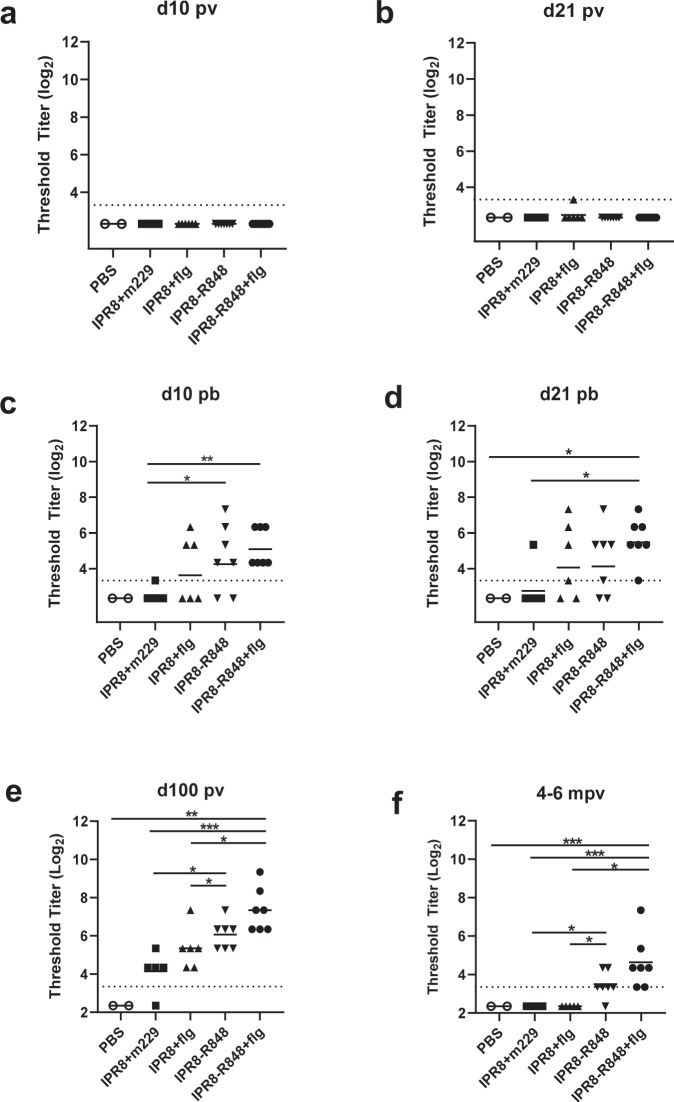
Vaccination with a TLRa adjuvanted vaccine elicits IgG to the HA stem. Newborn AGM received IPR8 with soluble flagellin (IPR8 + flg) (*n* = 6), conjugated to R848 (IPR8-R848) (*n* = 7), the combination of flagellin and IPR8-R848 (IPR8-R848 + flg) (*n* = 7), without functional adjuvant (IPR8 + m229) (*n* = 5), or PBS as a vehicle control (*n* = 3). Newborns were boosted with the same at d21 p.v. Plasma IgG titers to stabilized Ca09 HA stem were measured by ELISA at days 10 and 21 p.v. (**a**, **b**) or p.b. (**c**, **d**), approximately d100 p.v. (**e**) and 4-6 months (**f**) following initial vaccination. Data are shown as threshold titer (the lowest dilution at which sample OD was at least three times that of the assay background). The limit of detection (dotted line) is defined as the lowest sample dilution in the assay. Statistical significance was determined by ordinary one-way ANOVA with uncorrected Fisher’s LSD test for multiple comparisons. *p < 0.05, **p < 0.01. ***p < 0.001.

To investigate the ability of flagellin, R848, or the combination to elicit a sustained stem-specific antibody response, we measured plasma titers of stem-specific IgG in a cohort of mother-reared newborn NHP following vaccination using IPR8 with or without adjuvant. IgG specific to the HA A/California/4/2009 (Ca09) stem was not detectable by ELISA at day 10 postvaccination (p.v.), regardless of adjuvant strategy (Fig. 1a). At day 21 p.v., among newborns receiving adjuvanted vaccines, only a single animal (in the IPR8 + flg group) had detectable Ab capable of recognizing HA stem. Thus, a single dose of killed IAV vaccine did not readily elicit a detectable antibody response to the HA stem even in the presence of adjuvants (Fig. 1b).

Next, we investigated whether a secondary exposure to a TLRa adjuvanted vaccine at 21 days following initial vaccine administration was capable of inducing a detectable stem-specific antibody response. Groups vaccinated with IPR8 + flg or IPR8-R848 contained both responder and non-responder animals. Three of six (50%) newborns in the IPR8 + flg group and five of seven (71%) newborns in the IPR8-R848 group had detectable stem-specific IgG at day 10 post-boost (p.b.) (Fig. 1c). One additional IPR8 + flg vaccinated newborn became positive at d21 p.b. (Fig. 1d). All newborns receiving IPR8-R848 + flg displayed a detectable IgG response to the HA stem at both d10 and d21 p.b. In contrast, only one animal receiving IPR8 + m229 had stem-specific IgG titers at or above the limit of detection. Interestingly, both groups receiving IPR8-R848 and IPR8-R848 + flg had a significant increase in stem-specific IgG compared to the non-adjuvanted group at day 10 p.b., while only animals receiving IPR8-R848 + flg maintained significantly higher titers at day 21 p.b. These data show that inclusion of adjuvants can drive the production of antibodies to HA stem and that a major effect of adjuvants was the increased proportion of newborns with detectable stem-specific antibody.

### Inclusion of R848 adjuvant prolongs the presence of circulating stem-specific Abs

The newborn immune system is particularly challenged in the development of lasting immunity, i.e., Ab titers often wane rapidly compared to adults^59^. The presence of sustained Ab responses is dependent on development of LLPC. The process of selection, maturation, and differentiation in the GC can continue for weeks to months following antigen encounter. Thus, we measured plasma IgG to stem at later times following initial vaccination (~100 days and 4-6 months). Unexpectedly, given the findings at d21 p.b., all vaccinated animals, exempting one, that had received IPR8 + m229 had detectable IgG to the HA stem at this timepoint (Fig. 1e). Thus, the response measured at d21 p.b. did not fully predict the presence of stem-specific IgG generated following vaccination.

Although all but one infant had detectable stem-specific Ab, the amount present at d100 p.v. was dependent on the adjuvant. Animals receiving the IPR8-R848 + flg showed the highest amount of stem-specific IgG, although animals administered IPR8-R848 alone also displayed a significant increase compared to the IPR8 + m229 group (Fig. 1e). It was notable that the presence of flagellin did not result in significant increases in stem-specific antibody compared to the non-adjuvanted vaccine at this timepoint (Fig. 1e). Together, these data show that expansion of stem-specific IgG continues beyond d21 p.b., even in newborns receiving IPR8 without adjuvant. With that said, administration of R848 alone or co-administration of R848 and flagellin results in a significantly more robust response to the subdominant HA stem.

To determine whether the response had reached its peak by this time point, we followed this cohort of NHP out to 4-6 months from their initial vaccination as newborns and assessed plasma titers of stem-specific IgG (Fig. 1f). None of the animals that had received either IPR8 + m229 or IPR8 + flg had stem-specific IgG titers above the limit of detection, despite all animals having detectable antibody to whole PR8 at this time^52,53^. All animals that received the dual adjuvant and all but one receiving R848 alone retained detectable levels of stem-specific IgG.

**Fig. 2:**
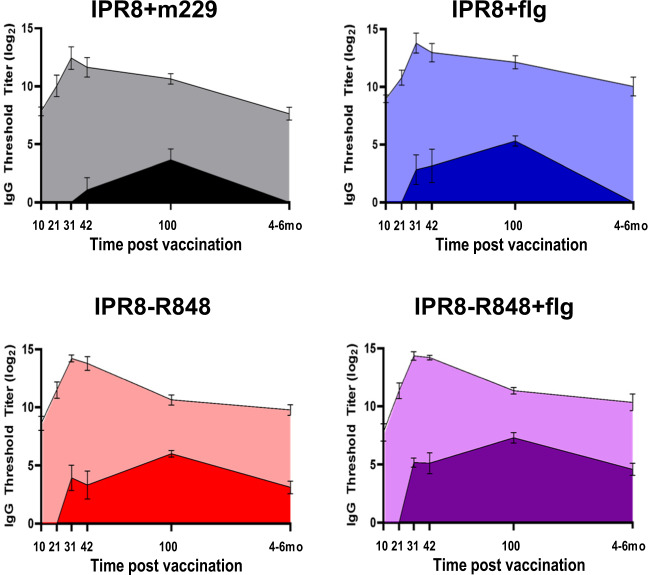
Kinetics of the IgG response to whole virus and HA stem. Average IgG titers over time to the HA stem (dark shading) and PR8 virion (light shading) for animals vaccinated with IPR8 + m229, IPR8 + flg, IPR8-R848, and IPR8-R848 + flg as in Fig. 1. Titers to PR8 have been previously reported for this cohort of animals (1, 2).

Our previous analyses showing that the TLRa adjuvants increased the magnitude and duration of antibody responses to the PR8 virion compared to vaccination with IPR8 alone^52,53^ raised the possibility that the increase in stem-specific IgG associated with these adjuvants was simply the result of global improvements in the humoral response rather than a mitigation of immunodominance. To explore this possibility, we compared the IgG response to the HA stem with our previously determined virus-specific IgG titers (Fig. 2)^52,53^. Although differences in assay methodology prevent quantitation of stem-specific antibody as an absolute proportion of the total response, this facilitates the visualization and comparison of the kinetics of the antibody response to HA stem versus to whole virion. While vaccination with IPR8 + flg, IPR8-R848, or IPR8-R848 + flg all elicited increased IgG titers to whole PR8 at 4-6 months compared to vaccination without adjuvant, the magnitude of the response was similar across adjuvant groups (Fig. 2). This is in contrast to the clear differences in the amount of stem-specific IgG present across the different adjuvant conditions, where the inclusion of R848 or both adjuvants resulted in sustained responses. Interestingly, titers to whole PR8 peaked at day 10 p.b. while levels of stem-specific IgG continued to rise through day 100 p.v. across all groups. This divergence between antibody to PR8 and the stem region suggests that the continued evolution of the antibody response to stem in animals given IPR8-R848 or IPR8-R848 + flg is not merely the result of a global increase in virus-specific Ab, but a specific improvement in the stem-specific response. Further, these data demonstrate differences in the kinetics of the stem-specific versus overall response. Finally, these results suggest that while both TLR5 and TLR7/8 are able to facilitate differentiation of stem-specific B cell clones into antibody secreting cells, TLR7/8 may enhance the ability of these clones to adopt and maintain a LLPC fate.

### The choice of TLRa adjuvant influences the avidity of stem-specific antibody present at late times following vaccination

**Fig. 3:**
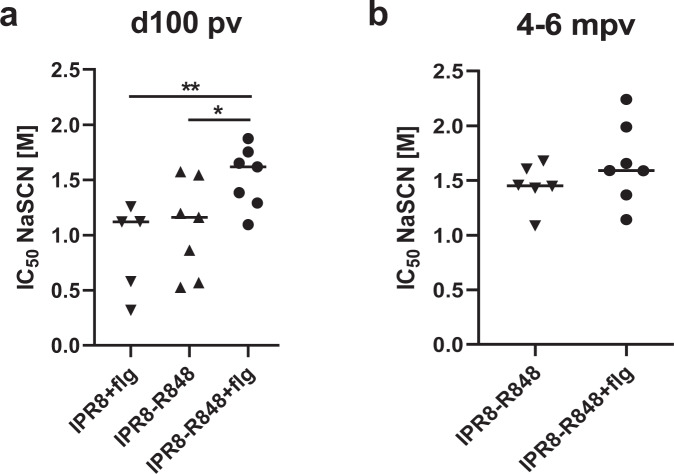
Adjuvant selection regulates avidity maturation of stem-binding IgG. The average avidity of Ca09 stem-specific IgG at day 100 p.v. (**a**) and 4-6 months p.v. (**b**) was calculated by determining the NaSCN concentration that resulted in a 50% reduction in absorbance compared to the untreated sample. Statistical significance was determined by ordinary one-way ANOVA with uncorrected Fisher’s LSD test for multiple comparisons (**a**) or unpaired *t*-test (**b**). *p < 0.05, **p < 0.01.

Increased antibody avidity, an outcome of somatic hypermutation and selection in the GC^60^, is associated with improved function in vivo. We postulated that in addition to increasing the total amount of stem-specific Ab at later times following vaccination, the presence of R848 may lead to greater avidity relative to that of flagellin. Ab avidity in non-adjuvanted animals was not assessed due to low titers and sample availability. Sensitivity to dissociation by treatment with chaotropic agents, which correlates with antibody off-rate^46,61^, was used to assess avidity. Animals assessed were those where stem-specific antibody was present at levels adequate for this analysis. We found no difference at ~d100p.v. in average antibody avidity between groups administered IPR8 with either R848 or flagellin despite significant differences in antibody titer (Fig. 3a). However, animals receiving IPR8-R848 + flg exhibited higher avidity stem-specific IgG than the other groups at this timepoint (Fig. 3a). By the 4–6 month timepoint, antibody avidity was similar in the IPR8-R848 and IPR8-R848 + flg groups, the only animals where antibody remained detectable (Fig. 3b). The similarity in avidity at this timepoint was the result of an increase in the avidity of IgG to stem in the R848 group from d100 to the 4-6 month timepoint (*p* = 0.03). There was no change in average antibody avidity of the HA stem Ab in animals administered IPR8-R848 + flg over this time (*p* = 0.22).

### The presence of adjuvants is associated with more IL-21 producing cells in the draining LN following challenge of vaccinated newborns

The sustained presence of affinity-matured stem-specific IgG in animals receiving IPR8-R848 with or without flagellin are consistent with a model wherein the adjuvants promote a more effective process of B cell differentiation and/or LLPC production. These processes primarily occur in the GC, where B cell differentiation is regulated by interaction with Tfh cells^43,44^. Tfh are critical for the differentiation of GC B cells towards a memory or LLPC fate and appear to have a strong influence on Ab responses following IAV vaccination and infection^62–64^. Additionally, Tfh have been implicated in the mitigation of immunodominance^65,66^. Given that newborns have reduced Tfh generation and function^28,30,34,67^, we hypothesized that modulation of Tfh cells by TLRa may account for the observed changes in generation of stem-specific Ab.

**Fig. 4:**
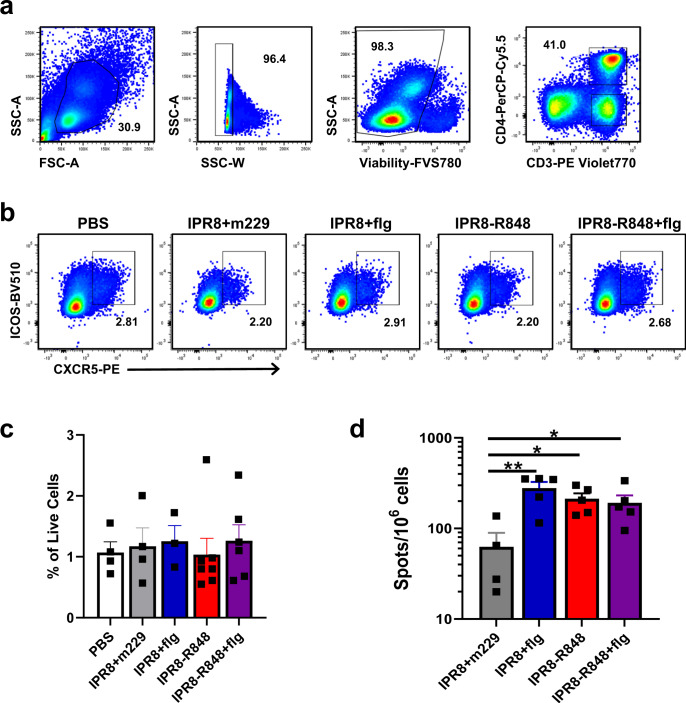
Inclusion of adjuvant does not result in detectable increases in the total number of Tfh in circulation at d10 p.b., but alters the quantity of influenza virus-specific Tfh in the draining lymph node following challenge. PBMC collected from newborn NHP at d10 p.b were assessed for the presence of circulating T follicular helper cells by flow cytometry. Three age-matched untreated controls were included in the PBS group to increase sample size. **a** Gating strategy to identify live CD4^+^ T cells. Tfh were defined as CXCR5^+^ ICOS^+^ cells within this population as shown by representative data in **b**. **c** Average frequencies of Tfh as a percentage of live cells in circulation. **d** A separate group of vaccinated infants was challenged by infection with 1 × 10^10^ EID50 of PR8 divided equally between the intranasal and intratracheal routes. On d14 p.c., tracheobronchial lymph nodes were isolated. IL-21 production was induced by culture in the presence of pooled peptides from the NA, HA, M1, and NP proteins (0.1 µg/ml for each peptide) for 48 h. IPR8 + m229 *n* = 4, adjuvanted vaccine groups *n* = 5. Statistical significance was determined by ordinary one-way ANOVA with uncorrected Fisher’s LSD test for multiple comparisons. *p < 0.05, **p < 0.01.

We first evaluated PBMC collected from vaccinated infants at d10 p.b. as the Tfh response would be expected to peak at approximately this time point after antigen encounter^68^. We chose this timepoint based on analyses in adult humans, but note it is possible that the kinetics of the response differs in young infants. Although the primary role of Tfh cells is in the GC, the number and phenotype of circulating Tfh (cTfh) has been shown to correlate with the GC Tfh response as well as the magnitude of the humoral response^62^. cTfh cells were identified as cells expressing both CXCR5 and ICOS within the CD3^+^CD4^+^ population (Fig. 4a, b). We did not find a difference in cTfh frequency in peripheral blood of infants receiving the various vaccines (Fig. 4c).

To gain further insights into these cells, we next sought to measure the number of vaccine-specific cTfh using the highly sensitive ELISPOT approach for quantifying these cells by IL-21 production. We analyzed cells obtained on d21 p.b. due to the lack of sample remaining from the d10p.b. timepoint. Cells were stimulated with pooled peptides from the HA, NA, NP, and M1 proteins of influenza virus as responses to these proteins account for majority of influenza-specific CD4^+^ T cells in humans^69^. IL-21 producing cells were below the limit of detection in these assays. Thus, we turned to an alternative approach, quantifying influenza-specific cells following challenge, that we had previously used in assessing IFNγ and IL-4 producing T cells^54^. In a distinct cohort of vaccinated newborns, newborns similarly vaccinated to those described above were challenged with PR8. Lung draining tracheobronchial lymph nodes were isolated on d14 p.c. and cells cultured in the presence of the pooled peptides for analysis by ELISPOT. The data in Fig. 4d show that the presence of adjuvant results in a significant increase in the number of IL-21 producing cells present in the draining lymph node following challenge. No differences were observed across the adjuvant conditions.

### R848 is capable of inducing significant increases in maturation and cytokine production in newborn bone marrow derived DC (BMDC)

**Fig. 5:**
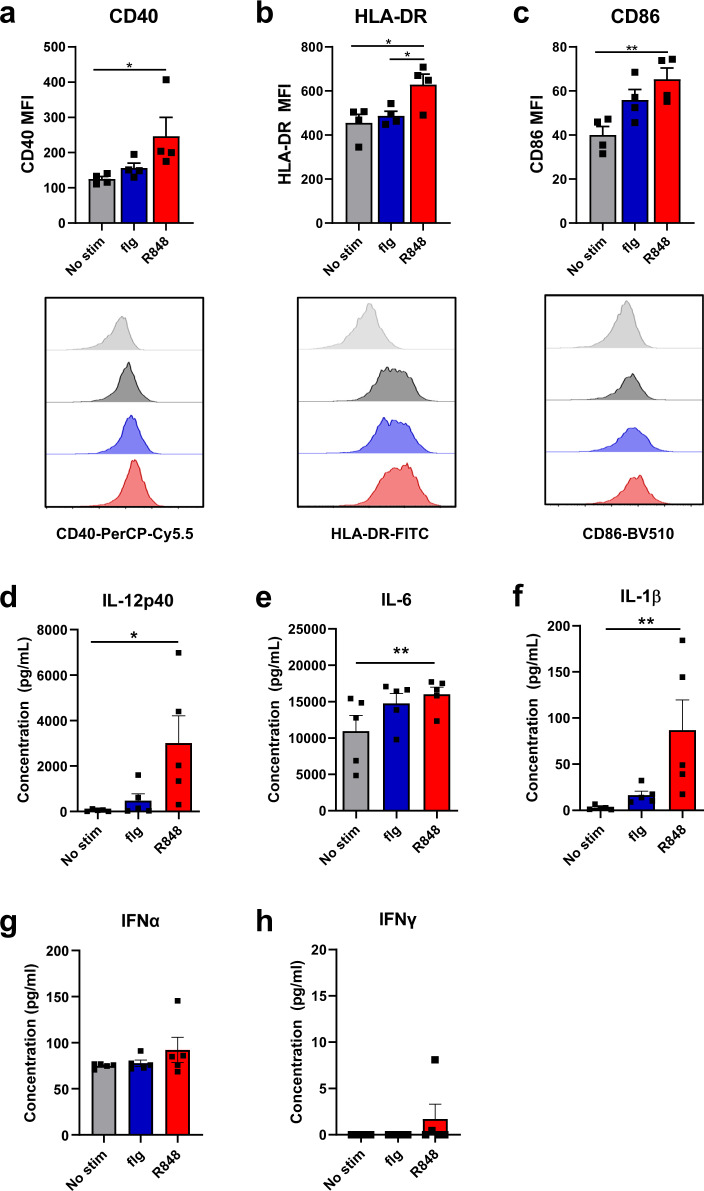
R848 induces robust activation of newborn BMDC. DC were differentiated from bone marrow obtained from naïve newborn AGM using hIL-4 and hGM-CSF. On day 6 of culture, cells were stimulated with R848, flagellin, or PBS for 24 h. CD11c^+^ positive cells were assessed for surface expression of CD40 (**a**), HLA-DR (**b**), and CD86 (**c**). Average median fluorescent intensity is shown with representative histograms below. Supernatants were harvested from flagellin or R848 stimulated BMDC cultures at 24 h. IL-12p40 (**d**), IFNα (**g**), and IFNγ (**h**) levels were assessed by ELISA. IL-6 (**e**) and IL-1β (**f**) were quantified by cytokine bead array. Statistical significance was determined using a Friedman test with uncorrected Dunn’s test for multiple comparisons. *p < 0.05, **p < 0.01.

Given the important role that DCs play in Tfh development^34,70,71^, we hypothesized that inclusion of TLRa adjuvants could improve Tfh and B cell function by increasing activation and cytokine production by newborn DC. To test this possibility, dendritic cells differentiated from the bone marrow of newborn AGM were stimulated in vitro with either flagellin or R848. Surface expression of maturation markers and production of cytokines were measured 24 h later. We found that R848 significantly increased the level of CD40, HLA-DR, and CD86 as well as production of multiple cytokines including IL-12p40, IL-1β, and IL-6. However IFNα or IFNγ were not significantly increased (Fig. 5). While flagellin stimulated BMDC showed increases in CD86 and IL-6, these did not reach statistical significance. Although not directly tested here, the capacity of R848 to increase maturation markers and pro-GC cytokine production in BMDC from newborns is consistent with a role for these APC in promoting an immune response that results in the persistence of HA stem-specific antibody in animals receiving an IPR8-R848 conjugated vaccine.

### TLRa adjuvants differentially impact Treg and Tfr phenotype

**Fig. 6:**
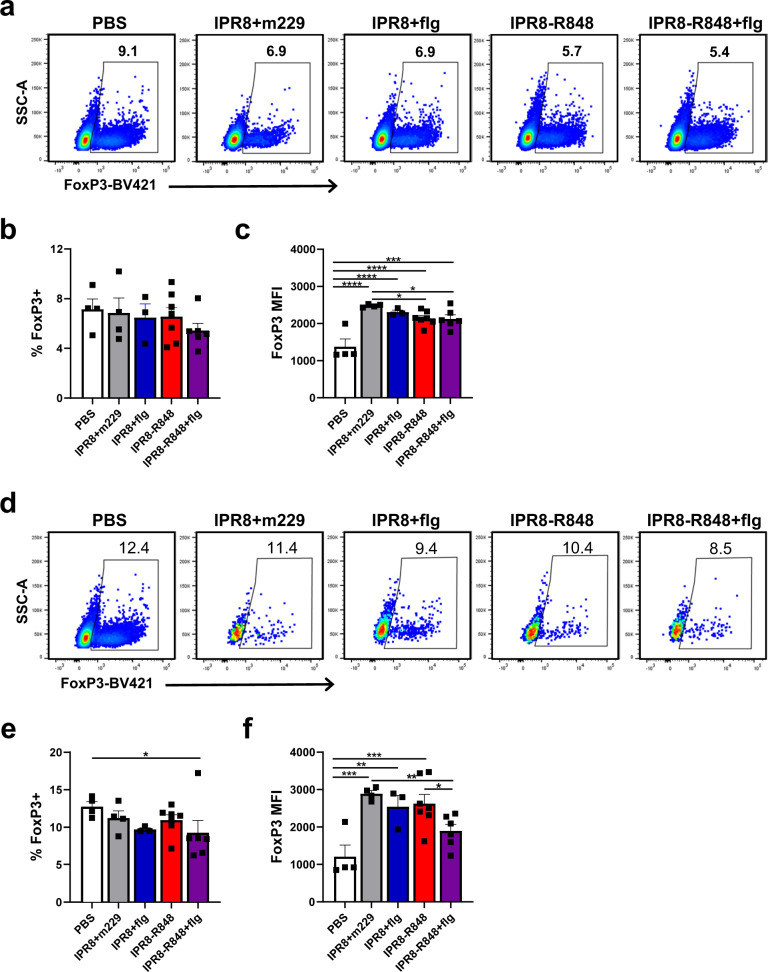
Vaccination in the presence of TLRa adjuvants alters Tregs in peripheral blood 10 days after vaccine boost. Tregs were quantified by intracellular staining for FoxP3 in peripheral blood collected from newborns at d10 p.b. or age matched naïve controls. **a** Representative data of FoxP3^+^ expression in live CD3^+^CD4^+^ PBMC. **b** Average percent of live CD3^+^CD4^+^ cells expressing FoxP3 in circulation. **c** Average median fluorescent intensity of FoxP3 within the CD3^+^CD4^+^FoxP3^+^ population. To assess the representation of Tfr within the T follicular subset, FoxP3^+^ cells were quantified within the CD3^+^CD4^+^CXCR5^+^ICOS^+^ population described in Fig. 4b; representative data are shown in **d** and average frequencies in **e**. **f** Average median fluorescent intensity of FoxP3 in the CD3^+^CD4^+^CXCR5^+^ ICOS^+^FoxP3^+^ Tfr population. Statistical significance was determined by ordinary one-way ANOVA with uncorrected Fisher’s LSD test for multiple comparisons. *p < 0.05, **p < 0.01, ***p < 0.001, ****p < 0.0001.

The increased representation of Tregs in newborns compared to their adult counterparts is another factor thought to contribute to diminished immune responses following vaccination early in life^36^. Tregs appear to play a role in the diversity and immunodominance hierarchy of Ab responses and have been shown to dampen the global Ab response to influenza vaccination^38,41,72^. To investigate whether the increased stem-specific Ab response seen with TLRa adjuvants is associated with changes in Tregs, we examined this population in PBMC collected from newborn animals at d10 p.b. The vaccine strategy used did not significantly alter the frequency of Tregs (Fig. 6a, b). However, inclusion of R848, regardless of the presence of flagellin, was associated with decreased expression of FoxP3 within the Treg population (Fig. 6c), a phenotype associated with reduced suppressive activity^73,74^.

We also assessed the presence of T follicular regulatory (Tfr) cells (CXCR5^+^CD4^+^FoxP3^+^) in light of recent studies exploring the suppressive activity of these cells in the GC response^75,76^. While all vaccine conditions trended towards a decreased Tfr frequency within the CXCR5^+^ICOS^+^ subpopulation compared to vehicle controls, this effect reached statistical significance only in the IPR8-R848 + flg group (Fig. 6d, e). The level of FoxP3 expression was also diminished in the animals receiving IPR8-R848-flg (Fig. 6f). We also evaluated the level of PD-1 and ICOS on various CD4^+^ T cell subsets. These markers are reported to increase with Treg activation state^77–80^. No significant changes were found in the level of these markers across vaccine groups for Treg or Tfr (Supplementary Figs. 1, 2). Of note, Tfh and Tfr exhibited higher expression of these markers compared to Tregs (Supplementary Figs. 1, 3).

## Discussion

Despite the shortcomings of current influenza vaccines, they remain the most effective way to reduce the morbidity and mortality associated with influenza virus infection. Because of this, it is essential that vaccines be able to confer protection to vulnerable populations such as newborns. In addition, protective responses that can work across influenza virus strains would be of particularly high benefit. Achieving these goals requires overcoming both the global reduction in immune responsiveness characteristic of early life as well as the immune system’s seemingly inherent preference for variable over conserved regions of the influenza HA protein. Here, we investigated whether inclusion of the TLR agonists flagellin and R848, either singly or in combination, as adjuvants could mitigate these barriers. We have used a nonhuman primate newborn model to probe these critical questions as this model is inarguably the most translatable to human newborns. As with humans, newborn NHP exhibit increased susceptibility to disease following influenza virus infection^81–83^. Anatomically the NHP lung is quite similar in structure to the human^84^ and there is a high degree of similarity between the NHP and humans in the distribution and responsiveness of TLR receptors^85^. Finally, the prolonged period of infancy and similarities in immune system maturity at birth in NHP provides a model where vaccination, boost, and challenge can be appropriately assessed in an infant.

Our previous studies have demonstrated that both flagellin and R848 can increase the magnitude and as a result persistence of the total antibody response to the influenza virion^52–54,86^. In the current study we found that the use of R848 or the combination of flagellin and R848 resulted in a significantly increased antibody response to the HA stem early that continued to increase at later times following vaccination. While flagellin provided some early benefit in a subset of newborns, this was not sustained. Changes in stem-specific antibody were associated with modulation of cells that regulate the antibody response and these alterations varied by adjuvant. Although we were unable to quantify antigen-specific cTfh following vaccination, newborns administered vaccines containing R848, flagellin, or their combination had higher numbers of influenza-specific Tfh following challenge compared to infants that were vaccinated in the absence of adjuvant. R848 was more effective at inducing activation and cytokine production in dendritic cells and reduced FoxP3 expression in Tregs. The combination of adjuvants was associated with decreases in both Tfr frequency and FoxP3 expression in Tfh and Tfr.

The ability of adjuvants to drive persistent antibody responses to HA stem in the newborn is a significant finding given the known hurdles associated with generating antibody to these subdominant epitopes and in establishing the LLPC niche in newborns^27,59^. Inclusion of R848 not only hastened the expansion of IgG to stem, as seen at d10 p.b., but also resulted in significant continued expansion of stem-specific IgG between d21 p.b. and 100 p.v. Although titers of stem-specific IgG declined in general between day 100 p.v. and 4-6 months p.v., the rate of decline was lower in animals receiving IPR8-R848. We speculate that the extended expansion and sustained response may be the result of improved and/or perhaps prolonged selection and differentiation of stem-specific LLPC clones in the GC. Extending the duration of GC activity has been reported to promote increases in neutralizing antibodies to subdominant epitopes in the setting of HIV^87,88^. Further, prolongation of the GC reaction can promote more extensive affinity maturation^89–91^.

Interestingly, we observed an increase in average antibody avidity of stem-specific IgG between d100 p.v. and 4-6 months in animals administered IPR8-R848, while antibody avidity in IPR8-R848 + flg group had already reached its maximum by d100 p.v. The continued increase in avidity in the IPR8-R848 vaccinated group resulted in similar avidity in R848 and dual adjuvanted recipients at the 4–6 month timepoint. This finding suggests the combination of flagellin and R848 may accelerate the process of affinity maturation. The continued increase in avidity in IPR8-R848 vaccinated newborns could reflect continued affinity maturation and export of plasma cells. However, the decrease in absolute stem-specific IgG titers between day 100 p.v. and 4-6 months in the IPR8-R848 group is also consistent with a model where the increase in avidity is due to preferential loss of lower-avidity antibody secreting cells over time.

Our data suggest the presence of R848 and/or flagellin results in an improved Tfh response as judged by the number present in the lung draining lymph node following challenge. We appreciate this is an indirect measure as it reflects expansion of memory cells following challenge, but does have benefit as it allows for assessment of IL-21 producing cells in the lymph node as opposed to circulation. Tfh number has been found to correlate with increases in antibody in a number of studies^68,92,93^. Further, there is evidence that Tfh can promote the generation of broadly reactive antibody in the context of SHIV_AD8_ infection of rhesus macaques^94^. Interestingly, the ability to facilitate broadly reactive antibody was dependent on the functional capabilities of elicited Tfh, in this case IL-4 production. The ability of our adjuvanted vaccines to impact Tfh function is an area that merits future study.

DC can exert a variety of regulatory effects on the humoral response including generation of Tfh^95,96^. While this makes DC an attractive target of vaccine strategies, newborn DC have diminished activation, maturation, and cytokine production in response to stimulation^28,97–99^. In our studies, we found R848 was superior to flagellin for inducing maturation and cytokine production in newborn BMDCs. R848 was effective in driving production of IL-12, one of the most notable impairments of newborn DC function^100–102^. This finding is consistent with the reported ability of TLR7/8 agonists to polarize mononuclear cells from human cord blood to a Th1 profile, counteracting the inherent Th2 bias of the neonatal immune system^103^. R848 can also increase GC formation and high-affinity Ab production in adult mice via DC-mediated B cell activation^104^. In contrast, TLR5 engagement was only able to promote a Th2-driven antibody response without affinity maturation^104^. Although the relationship between costimulatory signals on DC and antibody immunodominance has not yet been explored, further investigation is merited as decreased expression of CD80/CD86 on pulmonary DCs has been directly linked to altered immunodominance in CD8 cells in a newborn mouse model of RSV infection^105,106^.

Tregs provide suppressive feedback on the GC reaction and have been suggested to be a key component in maintaining immunodominance upon subsequent antigen exposures^41^. Their enhanced suppressive function in newborns as well as their established role in regulating immune responses makes them attractive targets for vaccines. The decrease we see in FoxP3 expression in Tregs supports the capacity of R848 conjugated vaccines to decrease Treg activity given the finding that FoxP3 levels are correlated with suppressive function^73,74^.

Generally, we did not observe measurable stem-specific antibody until after boost. While this may be the result of low levels of antibody, it is possible that after the priming dose, stem-specific B cell clones preferentially differentiate into memory B cells that rapidly differentiate into extrafollicular ASCs following boost. Indeed, clones with broad reactivity are more frequently found in memory B cell compartments than in plasma cell populations, and the reactivation of these clones provides protection during future encounters with heterologous strains of influenza^107–110^. The preference for memory differentiation may be attributable to the tendency towards a lower affinity that has been associated with polyreactivity^38^.

In summary, this study demonstrates that inclusion of TLR7/8 adjuvant R848 in an inactivated IAV vaccine can promote a lasting IgG response to the HA stem, while the TLR5 agonist flagellin produces a response that is diminished in both magnitude and persistence. Regardless of adjuvant, increased IgG to the HA stem appeared to be associated with improved Tfh responses. The presence of R848 was accompanied by Tregs with a dampened suppressive phenotype as well as improved maturation and cytokine production by newborn DC. Finally, our study reveals the unexpected finding that the kinetics of stem-specific Ab response is distinct from the overall Ab response to influenza virus following vaccination. These data provide new insights into the generation of stem-specific Ab and support pursuing development of an IAV vaccine that can effectively provide broad protection in young infants.

## Methods

### Animals

Newborn AGM used in this study were housed at the Wake Forest University School of Medicine African green monkey Research Colony. Newborns were mother-reared and housed in social groups throughout the course of the experiment except for the influenza virus challenged newborns, which were nursery reared.

### Vaccination and sampling

Newborn (4–6 days of age) received vaccines containing formalin-inactivated A/Puerto Rico/8/1934 (PR8) (H1N1) influenza virus with the following adjuvant conditions: R848 (*n* = 7), R848 and flagellin (flg) (*n* = 7), flg (*n* = 6), or inactive flagellin m229 (*n* = 5), which served as a non-adjuvanted vaccine. Two animals received PBS as vehicle controls. R848 was directly conjugated to IPR8 while flg and m229 were included in soluble form. Each vaccine contained 45 µg IPR8 administered intramuscularly into the deltoid muscle. Animals were boosted at day 21 post-vaccination (p.v.) with the same adjuvant formulation they received for their priming dose. Inactivation of PR8 was achieved by treating with 0.74% formaldehyde overnight at 37 °C. Virus was dialyzed against PBS and tested to assure the absence of infectivity. For the IPR8-R848 conjugate vaccine, an amine derivative of R848 was linked to SM(PEG)_4_ by incubation in DMSO for 24 h at 37 °C. R848-SM(PEG)_4_ was then incubated with influenza virus. Unconjugated R848 was removed by extensive dialysis. This construct was then inactivated by treatment with 0.74% formaldehyde overnight at 37°C, followed by dialysis. Successful conjugation was assessed by differential stimulation of RAW264.7 cells. To produce Salmonella enteritidis flagellin, E. coli BL21 (DE3) containing a pet29a::fliC encoding wild type flagellin or the truncated pet29a::229 encoding only the biologically inactive hypervariable region of flagellin were grown and lysates prepared in 8 M urea. Proteins were purified on Ni-NTA agarose. Endotoxin and nucleic acids were removed using an Acrodisc Mustang Q capsule. Purified proteins were extensively dialyzed against PBS. Peripheral blood was drawn by venipuncture at days 10 and 21 following vaccination and boost as well as at 100 days and 4-6 months after initial vaccination.

### Quantification of stem-specific IgG

To measure HA stem-specific antibody, plates were coated overnight with 5 ng of a headless A/California/4/2009 (H1N1) HA stabilized stem construct^111^. Plates were blocked for 1 h, after which they were washed with PBS + 0.01% Tween-20 (PBST). Plates were incubated for 3 h with serially diluted plasma. Starting dilutions were as stated. Plates were washed, incubated with HRP-conjugated goat anti-NHP IgG or IgM, and developed using TMB Ultra, after which the reaction was stopped with 0.1 N H_2_SO_4_. The absorbance for non-coated wells was subtracted for each animal, and threshold titer was defined as the lowest value that exceeded three times the average OD_450_ of the uncoated wells. For assessment of avidity, a sodium thiocyanate (NaSCN) dissociation step was included following sample incubation. Plasma was added at a single concentration selected for each animal based on the dilution that yielded 50% of the max OD_450_ in the ELISA to ensure consistent antibody amounts across varied antibody titers. Following binding, two-fold dilutions of NaSCN starting at 5 M were added to the plate for 15 min. Plates were then washed, incubated with HRP-conjugated goat anti-IgG, and developed as in the ELISA protocol. The IC_50_ was calculated using GraphPad Prism software.

### BMDC Culture and stimulation

Bone marrow was collected from newborn AGM (4-6 days old), purified by density gradient separation, and cryopreserved. For experiments, thawed cells incubated with 40 ng/ml human GM-CSF and 40 ng/ml human IL-4. On day 6, cells were stimulated with either 10 nM flagellin or 10 µM R848 for 24 h at 37 °C, after which cells and supernatants were harvested. Cells were stained with CD11c-PE (clone S-HCL-3), CD40-PerCP-Cy5.5 (clone 5C3), CD86-BV510 (clone 2331/(FUN-1)), and HLA-DR FITC (clone L243). Samples were acquired on a BD LSRFortessa X-20 and analyzed with FlowJo software.

### Cytokine quantification

IL-6 and IL-1β were assessed using a human inflammatory cytokine bead array (BD Biosciences) performed on supernatants collected from BMDC cultures per manufacturer’s protocol. Samples were acquired on a BD FACSCalibur; data were analyzed using FCAP Array software. IL-12p40, IFNγ and IFNα were quantified using a human ELISAs verified for NHP cross-reactivity.

### T cell flow cytometry

PBMC from newborns d10 p.b. were purified from peripheral blood by density gradient separation, aliquotted and stored in liquid nitrogen. For phenotyping by flow cytometry, cryopreserved cells were thawed in culture media as above and rested at 37 °C for 2 h. Cells were stained with Fixable Viability Stain 780 (BD Horizon). Surface staining was performed using the following antibodies: CD3-PE Violet 770 (clone 10D12), CD4-PerCP-Cy5.5 (clone L200), CD20-AlexaFluor 700 (clone 2H7), CXCR5-PE (clone MU5UBEE), PD-1-PE-Dazzle 594 (clone EH12.2H7), ICOS-BV510 (clone C398.4A). Cells were then washed, fixed and permeabilized with FoxP3/Transcription Factor staining kit, followed by FoxP3-BV421 (clone 206D). Samples were acquired on a BD LSRFortessa X-20 and analyzed with FlowJo software.

### T cell ELISPOT

A distinct cohort of vaccinated newborn AGM were challenged with PR8 (1 × 10^10^ EID50 divided equally between the intranasal (i.n.) and intratracheal (i.t.) routes, 0.25 ml i.t. and 0.25 ml i.n. (0.125 ml per nostril)) on d23-26 following boost. Lung draining tracheobronchial lymph nodes were isolated on d14 post challenge (p.c.) and stored in liquid nitrogen for future study. Thawed cells were cultured in the presence of pooled peptides from the NA (PR8), HA (PR8), M1 (A/California/04/2009), and NP (A/California/04/2009) proteins for 48 h in ELISPOT plates coated with anti-IL-21 antibody (human/NHP IL-21 ELISPOT) kit from MABTECH. Peptides were used at a final concentration of 0.1 µg/ml for each peptide. IL-21 was detected with the antibody provided in the human/NHP IL-21 ELISPOT kit from MABTECH. Plates were developed with BCIP/NBT-plus substrate solution. Plates were read using an ImmunoSpot Analyzer (Cellular Technology Ltd) and spot counts determined by analysis at ImmunoSpot.

### Statistics

All statistical analyses were performed using GraphPad Prism software. Statistical significance was determined by ordinary one-way ANOVA with uncorrected Fisher’s LSD test for multiple comparisons or Freidman test with uncorrected Dunn’s test for multiple comparisons as indicated. A two-tailed unpaired t test was used when two groups were compared. All titers were log_2_ transformed prior to statistical analysis.

### Study approval

The protocol was approved by the Wake Forest University School of Medicine IACUC and adhered to the U.S. Animal Welfare Act and Regulations.

### Reporting summary

Further information on research design is available in the Nature Research Reporting Summary linked to this article.

## Supplementary information

Supp Figures

REPORTING SUMMARY

## Data Availability

All data generated or analyzed during this study are included in this published article (and its supplementary files).
