# Supplementary material for: TLR agonists induce sustained IgG to hemagglutinin stem and modulate T cells following newborn vaccination
Source: NPJ Vaccines. 2022 Aug 29;7:102. doi: 10.1038/s41541-022-00523-8 (PMC9424286; doi:10.1038/s41541-022-00523-8)
Supplement: Supplementary file 1 — Supp Figures [file 41541_2022_523_MOESM1_ESM.pdf]

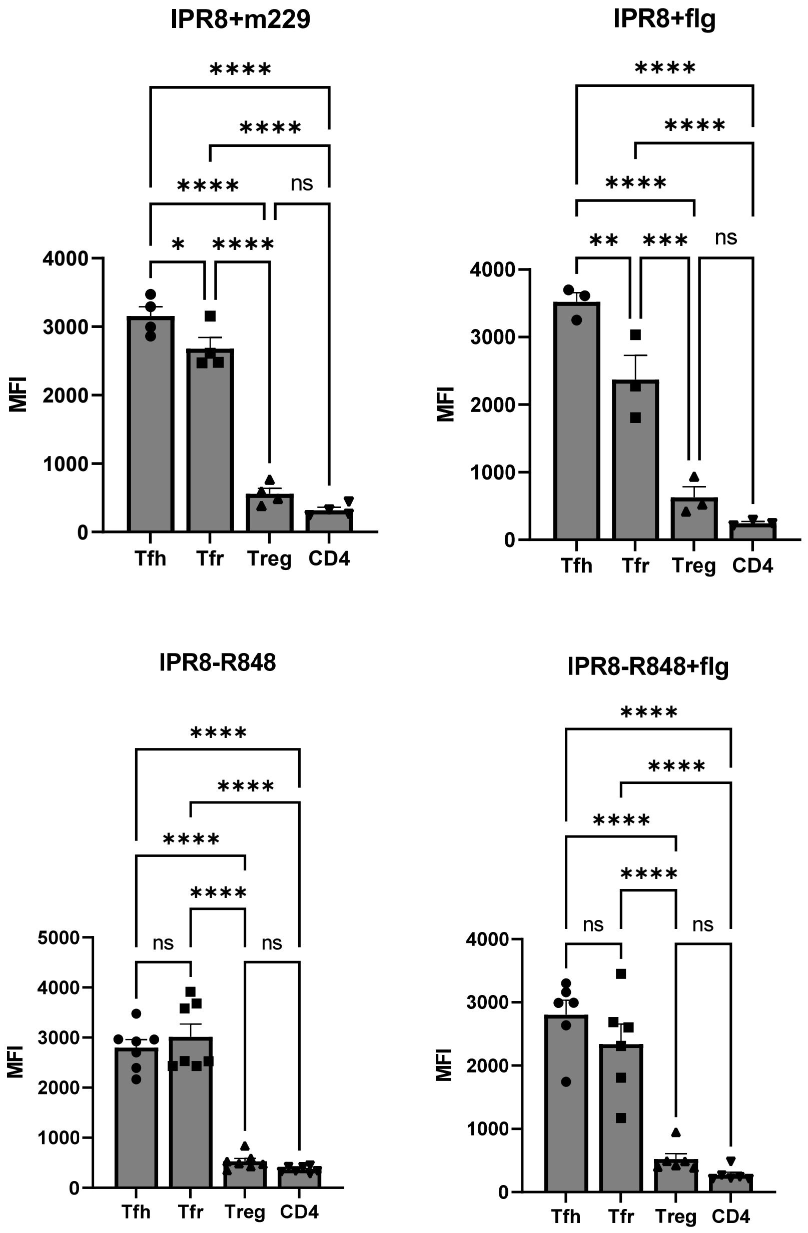

Supplementary Figure 1. PD-1 expression on CD4 subsets. PBMC collected from newborn NHP at d10 p.b were assessed for expression of PD-1 on Tfh, Tfr, Tregs, or CD4 cells that did not fall into these populations. Averages and SEM are indicated. Significance was determined by ANOVA. *p<0.05, **p<0.01, ***p<0.001, ****p<0.0001

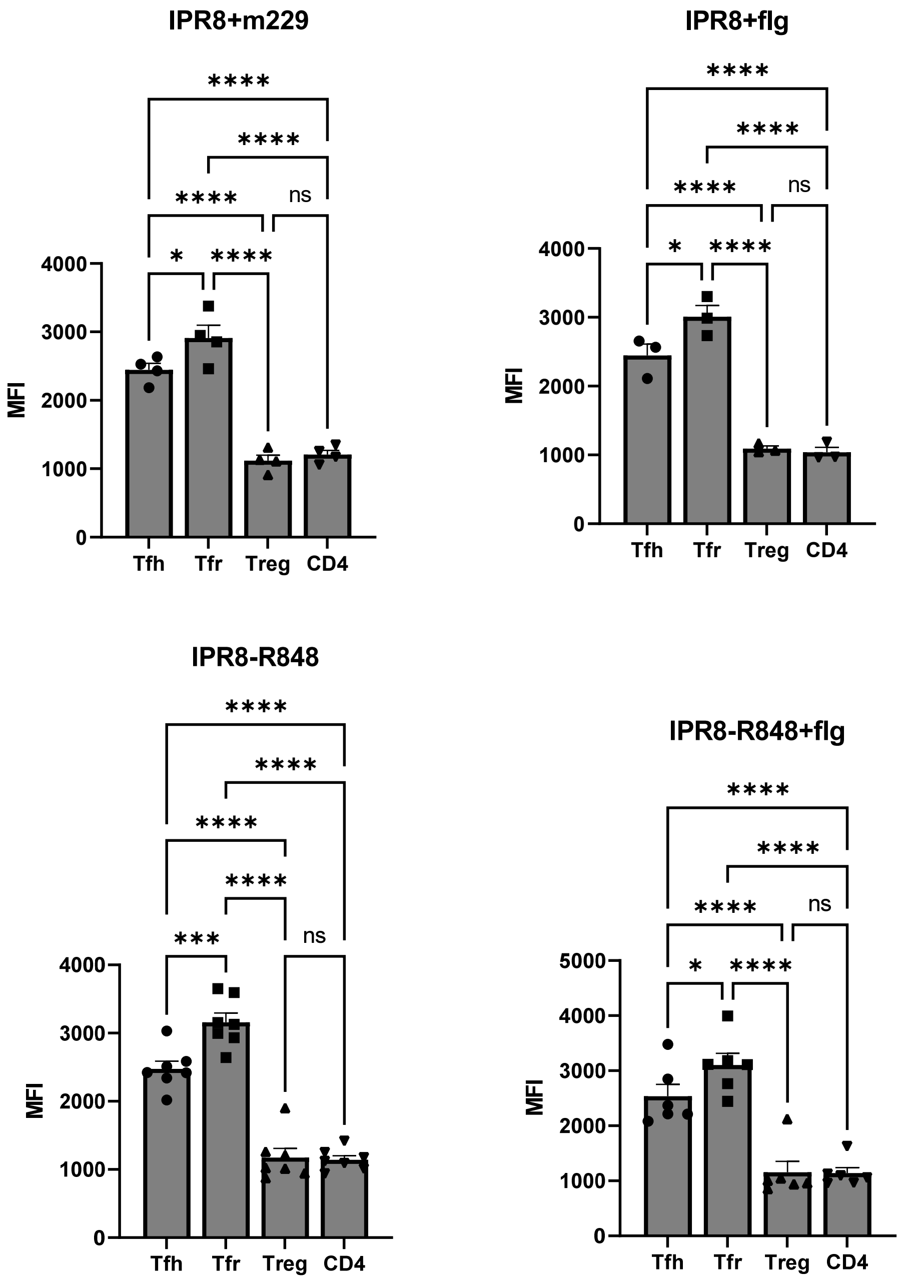

Supplementary Figure 2. ICOS expression on CD4 subsets. PBMC collected from newborn NHP at d10 p.b were assessed for expression of ICOS on T Tfh, Tfr, Tregs, or CD4 cells that did not fall into these populations. Averages and SEM are indicated. Significance was determined by ANOVA. *p<0.05, **p<0.01, ***p<0.001, ****p<0.0001
