# Supplementary material for: TLR agonists induce sustained IgG to hemagglutinin stem and modulate T cells following newborn vaccination
Source: NPJ Vaccines. 2022 Aug 29;7:102. doi: 10.1038/s41541-022-00523-8 (PMC9424286; doi:10.1038/s41541-022-00523-8)
Supplement: Supplementary file 2 — REPORTING SUMMARY [file 41541_2022_523_MOESM2_ESM.pdf]

nature portfolio

Corresponding author(s): Martha Alexander-Miller

Last updated by author(s): May 20, 2022

Reporting Summary

Nature Portfolio wishes to improve the reproducibility of the work that we publish. This form provides structure for consistency and transparency in reporting. For further information on Nature Portfolio policies, see our Editorial Policies and the Editorial Policy Checklist.

Statistics

For all statistical analyses, confirm that the following items are present in the figure legend, table legend, main text, or Methods section.

n/a	Confirmed	
☐	☒	The exact sample size (*n*) for each experimental group/condition, given as a discrete number and unit of measurement
☐	☒	A statement on whether measurements were taken from distinct samples or whether the same sample was measured repeatedly
☐	☒	The statistical test(s) used AND whether they are one- or two-sided<br>*Only common tests should be described solely by name; describe more complex techniques in the Methods section.*
☒	☐	A description of all covariates tested
☐	☒	A description of any assumptions or corrections, such as tests of normality and adjustment for multiple comparisons
☐	☒	A full description of the statistical parameters including central tendency (e.g. means) or other basic estimates (e.g. regression coefficient) AND variation (e.g. standard deviation) or associated estimates of uncertainty (e.g. confidence intervals)
☒	☐	For null hypothesis testing, the test statistic (e.g. *F*, *t*, *r*) with confidence intervals, effect sizes, degrees of freedom and *P* value noted<br>*Give P values as exact values whenever suitable.*
☒	☐	For Bayesian analysis, information on the choice of priors and Markov chain Monte Carlo settings
☒	☐	For hierarchical and complex designs, identification of the appropriate level for tests and full reporting of outcomes
☒	☐	Estimates of effect sizes (e.g. Cohen's *d*, Pearson's *r*), indicating how they were calculated

Our web collection on statistics for biologists contains articles on many of the points above.

Software and code

Policy information about availability of computer code

Data collection	Flow cytometric data were collected with CellQuest Pro version 6.0 or FacsDiva version 8.0.1, ELISA data were collected with Gen5 version 1.11.
Data analysis	FCAP Array software was used to analyze CBA data. FlowJo version 10 was used for other flow cytometric data. Graphpad Prism version 9 was used for statistical anlayses

For manuscripts utilizing custom algorithms or software that are central to the research but not yet described in published literature, software must be made available to editors and reviewers. We strongly encourage code deposition in a community repository (e.g. GitHub). See the Nature Portfolio guidelines for submitting code & software for further information.

Data

Policy information about availability of data

All manuscripts must include a data availability statement. This statement should provide the following information, where applicable:

- Accession codes, unique identifiers, or web links for publicly available datasets
- A description of any restrictions on data availability
- For clinical datasets or third party data, please ensure that the statement adheres to our policy

Provide your data availability statement here.

Human research participants

Policy information about studies involving human research participants and Sex and Gender in Research.

Reporting on sex and gender	*Use the terms sex (biological attribute) and gender (shaped by social and cultural circumstances) carefully in order to avoid confusing both terms. Indicate if findings apply to only one sex or gender; describe whether sex and gender were considered in study design whether sex and/or gender was determined based on self-reporting or assigned and methods used. Provide in the source data disaggregated sex and gender data where this information has been collected, and consent has been obtained for sharing of individual-level data; provide overall numbers in this Reporting Summary. Please state if this information has not been collected. Report sex- and gender-based analyses where performed, justify reasons for lack of sex- and gender-based analysis.*
Population characteristics	*Describe the covariate-relevant population characteristics of the human research participants (e.g. age, genotypic information, past and current diagnosis and treatment categories). If you filled out the behavioural & social sciences study design questions and have nothing to add here, write "See above."*
Recruitment	*Describe how participants were recruited. Outline any potential self-selection bias or other biases that may be present and how these are likely to impact results.*
Ethics oversight	*Identify the organization(s) that approved the study protocol.*

Note that full information on the approval of the study protocol must also be provided in the manuscript.

Field-specific reporting

Please select the one below that is the best fit for your research. If you are not sure, read the appropriate sections before making your selection.

☒ Life sciences ☐ Behavioural & social sciences ☐ Ecological, evolutionary & environmental sciences

For a reference copy of the document with all sections, see nature.com/documents/nr-reporting-summary-flat.pdf

Life sciences study design

All studies must disclose on these points even when the disclosure is negative.

Sample size	Vaccination groups included 3-7 animals based on power calculations guided by previous results.
Data exclusions	No data were excluded
Replication	Replicates were in the form of individual animals. Each animal in the group served as a replicate. Groups contained 3-7 animals/vaccine.
Randomization	Animals were randomly assigned to vaccine groups
Blinding	Blinding was not possible, but objective parameters were used as primary read-outs.

Reporting for specific materials, systems and methods

We require information from authors about some types of materials, experimental systems and methods used in many studies. Here, indicate whether each material, system or method listed is relevant to your study. If you are not sure if a list item applies to your research, read the appropriate section before selecting a response.

Materials & experimental systems

n/a	Involved in the study	
☐	☒	Antibodies
☒	☐	Eukaryotic cell lines
☒	☐	Palaeontology and archaeology
☐	☒	Animals and other organisms
☒	☐	Clinical data
☒	☐	Dual use research of concern

Methods

n/a	Involved in the study	
☒	☐	ChIP-seq
☐	☒	Flow cytometry
☒	☐	MRI-based neuroimaging

Antibodies

Antibodies used	Miltenyi<br>CD3-PE Violet 770 (clone 10D12)<br><br>BD<br>CD4-PerCP-Cy5.5 (clone L200)<br>CD20-AlexaFluor 700 (clone 2H7)<br>HLA-DR FITC (clone L243)<br>CD86-BV510 (clone 2331/(FUN-1))<br>CD11c-PE (clone S-HCL-3)<br><br>eBiocience<br>CXCR5-PE (clone MU5UBEE)<br><br>BioLegend<br>PD-1-PE-Dazzle 594 (clone EH12.2H7)<br>ICOS-BV510 (clone C398.4A)<br>FoxP3-BV421 (206D)<br>CD40-PerCP-Cy5.5 (clone 5C3)
Validation	These are validated by the manufacturer.

Animals and other research organisms

Policy information about studies involving animals; ARRIVE guidelines recommended for reporting animal research, and Sex and Gender in Research

Laboratory animals	African green monkey 3-5 days of age
Wild animals	The study did not involve wild animals
Reporting on sex	Infants were assigned to vaccine groups randomly as they were born.
Field-collected samples	The study did not involve samples collected form the field.
Ethics oversight	The animal protocol was approved by the Wake Forest School of Medicine IACUC and adhered to the U.S. Animal Welfare Act and Regulations.

Note that full information on the approval of the study protocol must also be provided in the manuscript.

Flow Cytometry

Plots

Confirm that:

☒ The axis labels state the marker and fluorochrome used (e.g. CD4-FITC).

☒ The axis scales are clearly visible. Include numbers along axes only for bottom left plot of group (a 'group' is an analysis of identical markers).

☒ All plots are contour plots with outliers or pseudocolor plots.

☒ A numerical value for number of cells or percentage (with statistics) is provided.

Methodology

Sample preparation	Cell samples are from the designated tissue, prepared by making single cell suspension and stored in liquid nitrogen. Plasma is obtained via venipuncture and stored at -80C.
Instrument	Becton Dickinson FacsCalibur or Fortessa
Software	Flow cytometric data were collected with CellQuest Pro version 6.0 or FacsDiva version 8.0.1
Cell population abundance	Cell sorting was not used in the study.
Gating strategy	Gating was performed by FSC/SSC gating followed by a viability dye to determine live cells. Subset markers were then used to identify populations of interest. Positivity was based on isotype control antibody staining.

☒ Tick this box to confirm that a figure exemplifying the gating strategy is provided in the Supplementary Information.
